# Prevalence of pre-operative anxiety and associated risk factors among patients awaiting elective surgery in a tertiary care hospital

**DOI:** 10.12688/f1000research.136320.2

**Published:** 2023-12-12

**Authors:** Suman Prasad Adhikari, Bishnu Deep Pathak, Bhuwan Ghimire, Sunil Baniya, Prabhas Joshi, Pooja Kafle, Prawesh Adhikari, Aakanksha Rana, Laxmi Regmi, Bishal Dhakal, Nabin Simkhada, Om Prakash Tandon, Indra Dev Pathak, Namrata Mahara Rawal

**Affiliations:** 1 Department of Neuropsychiatry, Nepalese Army Institute of Health Sciences, Kathmandu, Bagmati province, 10160, Nepal; 2 College of Medicine, Nepalese Army Institute of Health Sciences, Kathmandu, Bagmati Province, 10160, Nepal; 3 Department of Oxford University Clinical Research Unit, Patan Academy of Health Sciences, Lalitpur, Bagmati Province, 26500, Nepal; 4 Karnali Province Hospital, Birendranagar, Surkhet, 21700, Nepal; 5 Department of Internal Medicine, Kathmandu University School of Medical Sciences, Kavrepalanchok, Bagmati Province, 45200, Nepal

**Keywords:** anxiety, surgery, elective surgical procedures, anesthesia

## Abstract

**Background:**

Patients undergoing surgery have a fear of anesthesia and surgical procedures that results in anxiety. The global incidence of pre-operative anxiety is estimated at 60–92%. Age, gender, education, marital status, type of family, type of anesthesia and surgery, and history of surgery are the contributing factors. High levels of anxiety during the pre-operative period has negative impacts on surgical outcomes. The main objective of this study was to find out the prevalence of pre-operative anxiety and associated risk factors in a hospital setting of a developing country.

**Methods:**

This was a single center, analytical, cross-sectional study conducted among the admitted patients scheduled for elective surgeries in a tertiary care hospital. Non-probability convenience sampling was adopted and a total of 205 cases were included. The researchers themselves collected the data on the day before surgery using questionnaires comprised of two parts: semi-structured questionnaires prepared via literature review and Amsterdam Pre-operative Anxiety and Information Scale (APAIS). Data were analyzed in SPSS version 23. Bivariate and multivariate analyses were performed appropriately.

**Results:**

The prevalence of pre-operative anxiety was 25.85%. The median anaesthesia related, surgery related, and total anxiety scores were 4.00, 5.00 and 9.00 respectively. Likewise, the median score of information desired component scale was 5.00. Different anxiety scores were positively correlated with the information desire component score. The patients living in a nuclear family (adjusted OR, 2.480; 95% CI, 1.272–4.837, p = 0.008) and those without past history of surgery (adjusted OR, 2.451; 95% CI, 1.107–5.424, p = 0.027) had approximately 2.5 times higher risk of having pre-operative anxiety compared to those from a joint family and those having past history of surgery respectively. Those receiving spinal anesthesia had approximately two times lower risk of anxiety (adjusted OR, 0.511; 95% CI, 0.265–0.985, p = 0.045).

**Conclusions:**

One fourth of the patients had pre-operative anxiety. Type of family, type of anesthesia and past history of surgery were found to be the independent predictors of anxiety.

## Introduction

Anxiety is defined as an uneasy feeling about something which is uncertain. ^1^ It is common in patients awaiting surgical procedures. ^2^ Patients undergoing surgery are afraid of anesthesia and its implications. This fear results in anxiety. ^3^^,^^4^ Globally, the incidence of pre-operative anxiety is reported to range from 60% to 92%. ^2^ High levels of anxiety during the pre-operative period have deleterious effects on intra-operative and post-operative care. ^5^

Anxiety causes variable responses in patients scheduled for surgery. These include tachycardia, hypertension, sweating, elevated body temperature, apprehension, increased mental tension and aggression. ^6^^,^^7^ Pre-operative anxiety has unfavourable effects on induction and maintenance of anesthesia. Anxious patients require larger doses of anesthetic drugs and may have autonomic fluctuations as well. ^8^^–^^10^ Anxiety aggravates perception of pain and increases the need for post-operative analgesia. It delays recovery and lengthens the hospital stay. It has been found that such patients have increased nausea and vomiting, and higher risk of infections during the post-operative period. ^7^^–^^9^ There are several factors that contribute to significant levels of pre-operative anxiety. Some of these are age, gender, level of education, marital status, economic status, type and extent of surgery planned, past surgery and anaesthesia exposure, personal susceptibility and tolerance to stress, social security and existing psychiatric disorders. ^1^^,^^2^

It is obvious that pre-operative anxiety adversely affects the overall surgical outcomes and patients’ satisfaction. Hence, it should be addressed in the right way. Assessment of anxiety before surgical procedures is therefore very important. Likewise, a very few studies have been conducted on this topic in Nepal. The main objective of this study was to find out the prevalence of pre-operative anxiety in adult patients scheduled for elective surgery, and its associated risk factors in our setting.

## Methods

### Study setting

This study was conducted in the surgery ward of Shree Birendra Hospital, a tertiary care hospital of Nepal from the beginning of May 2022 till mid-October 2022. It is a teaching hospital of the Nepalese Army Institute of Health Sciences, College of Medicine, Kathmandu, Nepal. The ward consisted of two units: male and female, with a total of 150 beds. All the cases scheduled for elective surgeries are admitted here after surgical consultation.

### Study design and participants

This was a single-center, analytical, cross-sectional study conducted in elective surgery patients admitted to the surgery ward. Adult patients more than 18 years old who were scheduled for elective surgery under spinal or general anesthesia were included. Patients aged less than 18 years, with known psychiatric disorders under medication, and who could not understand Nepali language well were excluded. The included patients were scheduled for different major surgeries like gastrointestinal, hepato-biliary, urological and orthopedic surgeries.

### Sampling and sample size

Non-probability convenience sampling method was adopted. All the pre-operative patients in the surgery ward were taken according to their admission to the hospital for major elective surgeries.

The minimum sample size was calculated by using Cochran’s formula as follows:

$$n=Z^{2}\mathrm{pq}/e^{2}=\left(1.96^{2}\times 0.31\times 0.69\right)/0.07^{2}=167.34$$

Where:

n = sample size

Z = 1.96 at 95% confidence interval

p = Prevalence from previous study (prevalence of pre-operative anxiety in reference no. 1 study is 31%) ^1^

q = 1 – p = 0.69

e = standard error (taking 7%)

The calculated minimum sample size was approximately 167. However, we took 205 cases in our study.

### Data collection and study variables

The researchers approached the patients’ ward one day before surgery. Written informed consent from the pre-operative patients who were willing to participate in the study was obtained after explaining the research objectives and processes in detail. Then, the researchers asked the patients questions while they were comfortably seated or lying down.

The questionnaires were comprised of two parts. Part-I contained semi-structured questions prepared through extensive literature review. These included socio-demographic variables like age, gender, religion, profession, education, type of family and marital status. Likewise, it also incorporated clinical and surgical characteristics that could possibly affect pre-operative anxiety levels like presence of co-morbidities, duration of hospital stay before surgery, type of surgery and anesthesia, past history of major surgery. Similarly, part-II included the Amsterdam Pre-operative Anxiety and Information Scale (APAIS) ^11^^–^^14^ which contained six questions in total. Two questions were related to patients’ anxiety about surgical procedures, the next two questions concerned anxiety of anesthesia, and the remaining two questions evaluated the need for information regarding surgery and anesthesia. Each question was scored subjectively by the patient in a 5-point Likert scale graded from 1 through 5, where ‘1’ denotes ‘minimal’ or ‘not at all’ and ‘5’ denotes ‘extremely.’ A total anxiety score of more than 10 was considered having pre-operative anxiety. Likewise, in the information scale, a score of 2–4 was classified as having no or little information requirement, 5–7 as having an average information requirement and a score of 8–10 as having a high information requirement.

The reliability of APAIS in our study sample was high with Cronbach’s alpha = 0.852 (acceptable with >0.7). Before starting data collection, pre-testing was done in 10% of the study sample.

### Ethical consideration

Ethical approval was obtained from the Institutional Review Committee of the Nepalese Army Institute of Health Sciences (IRC Reg. No. 420, Ref No. 245). Before conducting the study, permission was obtained from the hospital authority and the Head of the Department of Surgery. Written informed consent was taken from the patients themselves. In case of uneducated patients, the investigators themselves explained the entire content of the consent form in their native language, and the consent was approved by taking their finger stamps. ^15^ The privacy and anonymity of patient information were well-maintained.

### Data analysis

Initially, the collected data was entered in Microsoft Excel, 2010 after which it was imported and analyzed in IBM SPSS (Statistical Package for the Social Sciences), version 23. The Shapiro-Wilk W test was performed to check the normality of continuous data. The median/interquartile range was calculated for non-normally distributed variables, which included age of patients, duration of hospital stay before surgery, pre-operative anxiety scores, and information desired component scores. The dependent variable was pre-operative anxiety (yes/no), while the rest of the variables influencing anxiety levels were independent variables. The categorical variables were expressed in frequency and percentages. First, Chi-square/Fisher’s exact test was applied to check the association between dependent and independent categorical variables. For continuous variables, a Mann Whitney U test was performed to check association. Thus, statistically significant variables showing no collinearity among themselves were further tested by performing binary logistic regression analysis. The significance level was taken as p <0.07, with a 95% confidence interval considering a 7% standard error throughout the analysis. Likewise, the Spearman’s correlation between different anxiety scores and information desire component scores was also calculated.

## Results

### Socio-demographic characteristics

A total of 205 cases were taken and analyzed. Among them, 105 (51.22%) were males and 100 (48.78%) were females. The overall median age was 47 (34–59) years out of which 108 (52.68%) patients belonged to age groups less than or equal to 50 years. One hundred and two (49.76%) patients lived in a nuclear family whereas the rest (50.54%) were from a joint family. Most of the patients followed Hinduism (86.83%) followed by Buddhism (11.71%) and Islam (1.46%). Regarding occupation, only nine (4.39%) were health professionals. Most of the participants (87.32%) were educated to different academic levels, i.e., primary or secondary or above secondary. One hundred and seventy (82.93%) were married, 19 (9.27%) were unmarried and 16 (7.81%) were widows/widowers ( Table 1). The full dataset can be found under underlying data. ^16^

**Table 1.** Factors affecting pre-operative anxiety in elective surgery patients.

SN	Variables	Total n (%)	Pre-operative anxiety		p-value
			Yes n (%)	No n (%)	
1	Age category (years)				0.325
	≤ 50	108 (100.00)	31 (28.70)	77 (71.30)	
	> 50	97 (100.00)	22 (22.68)	75 (77.32)	
2	Gender				0.554
	Male	105 (100.00)	29 (27.62)	76 (72.38)	
	Female	100 (100.00)	24 (24.00)	76 (76.00)	
3	Occupation				0.698
	Health personnel	9 (100.00)	3 (33.33)	6 (66.67)	
	Non-health personnel	196 (100.00)	50 (25.51)	146 (74.49)	
4	Education				0.726
	No education	26 (100.00)	7 (26.92)	19 (73.08)	
	Primary	24 (100.00)	8 (33.33)	16 (66.67)	
	Secondary	93 (100.00)	21 (22.58)	72 (77.42)	
	Above secondary	62 (100.00)	17 (27.42)	45 (72.58)	
5	Type of family				**0.006**
	Nuclear	102 (100.00)	35 (34.31)	67 (65.69)	
	Joint	103 (100.00)	18 (17.48)	85 (82.52)	
6	Marital status				0.084
	Unmarried	19 (100.00)	2 (10.53)	17 (89.47)	
	Married	170 (100.00)	44 (25.88)	126 (74.12)	
	Widow/widower	16 (100.00)	7 (43.75)	9 (56.25)	
7	Type of anesthesia				**0.044**
	General	88 (100.00)	29 (32.95)	59 (67.05)	
	Spinal	117 (100.00)	24 (20.51)	93 (79.49)	
8	Past history of surgery				**0.044**
	Yes	61 (100.00)	10 (16.39)	51 (83.61)	
	No	144 (100.00)	43 (29.86)	101 (70.14)	
9	Type of surgery				0.507
	Gastrointestinal	71 (100.00)	22 (30.99)	49 (69.01)	
	Hepato-biliary	55 (100.00)	14 (25.45)	41 (74.55)	
	Urology	44 (100.00)	8 (18.18)	36 (81.82)	
	Orthopedics	35 (100.00)	9 (25.71)	26 (74.29)	
10	Duration of hospital stay before surgery (days)	3.00 (1.00-4.00)	3.00 (1.00-5.00)	2.00 (1.00-4.00)	0.164

p-value is obtained by Chi-square/Fisher’s exact test for categorical variables and Mann Whitney U test for continuous variable.

### Clinical and surgical characteristics

The most common co-morbidity was hypertension (28.29%) followed by diabetes mellitus (12.20%). All these co-morbidities were well controlled, and the patients were well optimized in their pre-operative period. Most of the cases (34.63%) were undergoing gastrointestinal surgery followed by hepato-biliary (26.83%), urology (21.46%) and orthopedic surgery (17.07%). Among them, 61 (29.76%) had a past history of surgery performed under spinal or general anesthesia. Among the cases undergoing these surgeries, the majority were planned to receive spinal anesthesia (57.07%), and the remaining 88 (42.93%) would be operated on under general anesthesia ( Table 1).

### Pre-operative anxiety: Prevalence and scores

Out of the total pre-operative cases, 53 (25.85%) had pre-operative anxiety (i.e. a total anxiety score ≥11). The median anesthesia related, surgery related, and total anxiety scores were 4.00, 5.00 and 9.00 respectively. Likewise, the median score of information desired component scale was 5.00. Seventy-two (35.12%) patients had little or no information requirement regarding the surgical procedure and/or anesthesia, 101 (49.27%) had an average information requirement, and 32 (15.61%) had a high information requirement. There was a statistically significant positive correlation between different anxiety scores and the information desired component score. The correlation was low positive between information desired component and anaesthesia/surgery related anxiety scores whereas it was moderately positive with combined anxiety score ( Table 2).

**Table 2.** Correlation between different anxiety scores and information desire component score.

	Anaesthesia related anxiety	Surgery related anxiety	Combined anxiety component	Information desired component
Median score	4.00	5.00	9.00	5.00
IQR	3.00 – 5.00	4.00 – 6.00	7.00 – 11.00	4.00 – 6.00
Spearman’s correlation coefficient (ρ)	**0.494 (p < 0.001)**	**0.427 (p < 0.001)**	**0.515 (p < 0.001)**	

IQR: Interquartile range.

### Factors affecting pre-operative anxiety

The bivariate analyses showed that the patients living in a nuclear family had significantly higher pre-operative anxiety compared to those from a joint family (35[34.31%] vs 18[17.48%], p = 0.006). Likewise, the patients who were to receive general anesthesia for their surgeries reported a significantly higher anxiety level than those receiving spinal anesthesia (29[32.95%] vs 24[20.51%], p = 0.044]. Similarly, the patients who had a past history of surgery were significantly less anxious during the pre-operative period than those with no significant past surgical history (10[16.39%] vs 43[29.86%], p = 0.044). All other parameters did not show any significant difference in pre-operative anxiety in surgical patients.

### Logistic regression

The binary logistic regression analysis showed that the patients who were living in a nuclear family had approximately 2.5 times higher risk of having pre-operative anxiety compared to those living in a joint family (adjusted OR, 2.480; 95% CI, 1.272–4.837, p = 0.008). Likewise, the cases who were going to receive spinal anesthesia had approximately two times lower risk of anxiety in the pre-operative period than those undergoing general anesthesia (adjusted OR, 0.511; 95% CI, 0.265–0.985, p = 0.045). Similarly, the patients who did not have a past history of surgery were approximately 2.5 times more likely to have pre-operative anxiety in comparison to those who had some surgery in the past (adjusted OR, 2.451; 95% CI, 1.107–5.424, p = 0.027). In this way, type of family, type of anesthesia and past history of surgery (yes/no) were found to be the independent predictors of pre-operative anxiety ( Table 3).

**Table 3.** Binary logistic regression showing independent predictors of pre-operative anxiety.

Variables	AOR	[95% C.I.]		Std err.	p-value
Type of family					
Joint	1 (Reference)				
Nuclear	2.480	1.272	4.837	0.341	**0.008**
Type of anesthesia					
General	1 (Reference)				
Spinal	0.511	0.265	0.985	0.335	**0.045**
Past history of surgery					
Yes	1 (Reference)				
No	2.451	1.107	5.424	0.405	**0.027**

CI: Confidence interval; AOR: Adjusted odds ratio; Std Err: Standard error.

## Discussion

In our study, the prevalence of pre-operative anxiety was 25.85%, with average total anxiety score being 9.00 and information desired component score being 5.00. Almost half of the patients (49.27%) had an average information requirement, and 15.61% had a high information requirement. The anxiety scores and information scale score were significantly positively correlated. This infers that the patients with higher pre-operative anxiety were in the need of more information and counseling regarding their surgical procedures. The patients living in a nuclear family, not having a past history of surgery, and scheduled to receive general anesthesia had 2 to 2.5 times higher risk of having anxiety during the pre-operative period compared to those from joint family, those having past surgical history and those going to receive spinal anesthesia.

The prevalence of pre-operative anxiety in our study is comparatively lower than that of worldwide data that estimates its incidence to be around 60 to 92%. ^2^ A study done in Ethiopia showed that significant anxiety was present in 70.3% of the patients scheduled for surgery. ^8^ Likewise, in Saudi Arabia, 60% of the pre-operative patients had high anxiety. ^5^ Prevalence of anxiety was also found to be high (76.7%) amongst Sri Lankan patients admitted for surgery. ^3^ To the contrary, a study conducted in India depicted that overall prevalence of anxiety in elective surgical patients was 31%, which is comparable to our findings. ^1^ On the other hand, a similar study in Nepal showed the presence of severe pre-operative anxiety in the majority (57.3%) of surgical patients. ^2^ These differences could be due to the difference in sample size, sampling techniques, different study population and hospital settings, and different types of anxiety measuring tools being used in these studies worldwide. Moreover, we assessed the anxiety level on the day before surgery. Had we evaluated it on the day of surgery, the anxiety level would have been raised significantly.

Surgery is indeed a psychologically stressful experience. So, some degree of anxiety is natural to this unpredictable and potentially threatening situation. However, high anxiety during pre-operative period has negative impacts on surgical outcomes. ^5^ The patients start to have anxiety as soon as the surgery is planned, and it increases to a maximum on admission to hospital. ^6^ Anxiety causes unnecessary fear, irritability and autonomic fluctuations in admitted patients. These unpleasant symptoms may compel them to refuse the planned surgeries. ^8^ Pre-operative anxiety negatively affects induction of anesthesia. It causes different problems like difficult venous access, delayed jaw relaxation and coughing during induction. ^8^^,^^9^ It has been found that such patients have increased nausea and vomiting, and higher risk of infections during post-operative period. ^7^^–^^9^

Addressing patients’ anxiety during the pre-operative period is a must. ^3^ There are both pharmacological and non-pharmacological methods of reducing anxiety in the pre-operative period. Patients are routinely administered hypnotic/anxiolytic medications before surgery. Non-pharmacological methods include effective communication strategies, and provision of surgical information in videos or written form. ^17^ Past studies have found that patient education may decrease anxiety and reduce the need for sedation to relieve anxiety and pain. ^18^^–^^20^

There are a few limitations of this study to be mentioned. First, non-probability convenience sampling was adopted with a smaller sample size. Moreover, this is a single center study, so the findings may not be generalizable for a larger population or whole country. Future studies should include multiple study centers in different parts of the country with a relatively larger sample size. Next, anxiety level was measured at a single instance. However, past studies have shown that anxiety in pre-operative patients differs significantly in the perioperative period. Moreover, it would have been relevant if post-operative anxiety had also been studied. Likewise, pediatric patients were excluded from our study. Children also suffer significant anxiety during the pre-operative period, so including this group in the research would have increased the clinical relevance. In addition to this, we did not use the translated tool because none of the patients were educated, and a few of them could not read or write. To avoid this barrier, the researchers asked the participants questions in a standard way and assisted them to complete the questionnaires in an unbiased manner. APAIS has been validated in different countries ^21^^–^^26^ including South East Asia, so using this tool in our setting was considered to be appropriate.

Despite many limitations, our study provides some useful clinical information. First, it puts light on the anxiety burden among pre-operative patients in developing countries like ours. Next, it depicts the possible underlying risk factors contributing to significant anxiety during the pre-operative period and makes clinicians aware of the management of these factors beforehand. This adds to the existing medical knowledge, and possibly enhances patient care and satisfaction. Besides, this research encourages further analytical studies with a larger sample size and superior design to be conducted in the future. All these contribute to enhanced perioperative care and management of surgical patients in hospital settings.

## Conclusions

In our study, one fourth of the patients experienced anxiety during the pre-operative period. The majority of them had an average information requirement regarding the surgical procedure and anesthesia. Type of family, type of anesthesia and past history of surgery were the independent predictors of pre-operative anxiety. These factors should be assessed and addressed well before performing surgery.

## Consent

Written informed consent for publication of the participants’ details was obtained from the participants. In the case of uneducated patients, the consent was approved via taking their finger stamps after explaining all the contents of consent form in their native language.

## Data Availability

Figshare: Underlying data for ‘Prevalence of pre-operative anxiety and associated risk factors among patients awaiting elective surgery in a tertiary care hospital’, https://www.doi.org/10.6084/m9.figshare.23244059.v4. ^16^ This project contains the following underlying data:
• Data file 1: Excel data.xlsx • Data file 2: Preoperative data.sav Figshare: Extended data for ‘Prevalence of pre-operative anxiety and associated risk factors among patients awaiting elective surgery in a tertiary care hospital’, https://www.doi.org/10.6084/m9.figshare.23541501.v1. ^15^ This project contains the following extended data:
• Supplementary file 1: Questionnaire.pdf • Supplementary file 2: Consent form in English and Nepali.pdf Data are available under the terms of the Creative Commons Attribution 4.0 International license (CC-BY 4.0)
