# Supplementary material for: Prevalence of pre-operative anxiety and associated risk factors among patients awaiting elective surgery in a tertiary care hospital
Source: F1000Res. 2023 Dec 12;12:1207. Originally published 2023 Sep 25. [Version 2] doi: 10.12688/f1000research.136320.2 (PMC10839854; doi:10.12688/f1000research.136320.2)
Supplement: Peer review history

# Reviewer report: Reviewer response for version 2

**DOI:** 10.5256/f1000research.159965.r229544

**Published:** 2024-02-01

**Authors:** Suraj Shakya

**Affiliations:** 1 Department of Psychiatry and Mental Health, Institute of Medicine, Tribhuvan University, Kirtipur, Kathmandu, Nepal

Thank you for submitting the revised draft. The authors have integrated some suggestions, while some have not been included, possibly reflecting differences in opinion. Finally, I suggest only one specific correction: setting a significance level of p < 0.07 is unconventional and could elevate the risk of Type I error (false positives). Please take care of that.

Is the work clearly and accurately presented and does it cite the current literature?

Partly

If applicable, is the statistical analysis and its interpretation appropriate?

I cannot comment. A qualified statistician is required.

Are all the source data underlying the results available to ensure full reproducibility?

Partly

Is the study design appropriate and is the work technically sound?

Partly

Are the conclusions drawn adequately supported by the results?

Yes

Are sufficient details of methods and analysis provided to allow replication by others?

Yes

Reviewer Expertise:

I am an academic faculty and clinical psychologist, with research interest in psychometrics and psychological intervention.

I confirm that I have read this submission and believe that I have an appropriate level of expertise to confirm that it is of an acceptable scientific standard.

# Reviewer report: Reviewer response for version 1

**DOI:** 10.5256/f1000research.149457.r210142

**Published:** 2023-10-24

**Authors:** Suraj Shakya

**Affiliations:** 1 Department of Psychiatry and Mental Health, Institute of Medicine, Tribhuvan University, Kirtipur, Kathmandu, Nepal

The authors have undertaken an important study on pre-operative anxiety in a tertiary-level hospital in Kathmandu. Undoubtedly, this is an important research, and the write up is okay too. However, there are some points for improvement. Most of the points are minor comments, except one major comment: 

1. Heading: Consider removing the word 'prevalence' from the heading, as it appears fine without it. Just a suggestion only.  
2. Abstract: Include the number of participants in the methods section of the abstract.
3. Abstract: In the first sentence of the results section, present only percentages rather than raw numbers.
4. Abstract (Result): When stating, "The patients living in a nuclear family...approximately 2.5 times higher risk of having pre-operative anxiety," specify the reference group for comparison (e.g., 2.5 times higher risk than...?).
5. Introduction, 3rd paragraph, 4th sentence: Rephrase the word 'rarely' since there are some researches in Nepal as well.
6. Method, Study Design, 1st sentence: Reevaluate whether the design can be termed analytical; it seems more like a 'cross-sectional descriptive' design. Just my opinion, but please seek input from colleagues in this field.
7. Sampling and sample size: Please justify why this is consecutive sample (instead of purposive, or convenience method); and this statement is not clear 'all the patients were taken consecutively'.
8. Sampling and sample size (sample size calculation): I was curious, why were data from similar studies in Nepal not used? Not necessary to incorporate this suggestion, if the team think this is justified.
9. Data Collection and Study Variables, 2nd paragraph, 2nd sentence: Provide citations for the literature being referred to, if possible, to enhance authenticity.
10. Data Collection and Study Variables (this is a Major Comment): If translation was performed, particularly for illiterate participants, clearly describe the process. Please mention clearly how many enumerators were involved, and how did they maintain uniformity in instant translation during data collection, in method section (in data collection section). As you have mentioned 'standard way' (2nd last para of discussion section), please elaborate on what this means. This is very important; otherwise, this would seem a major flaw and limitation of this study. 
11. Data Analysis: Confirm whether the significance level is set at 0.05 or 0.07.
12. Results, Socio-Demographic/Surgical Characteristics: Simplify and condense the wording in this section, as detailed information may be found in the tables. For instance, this sentence 'Of these, 108 (52.68%) and 97 (47.32%) belonged to age groups less than or equal to 50 years and above 50 years respectively.' could be deleted. 
13. Ensure uniformity in the presentation of numbers and percentages [3, 5% vs 3(5%)] throughout the entire article, especially in the results section.
14. Table 1: Remove the '(100.00)' in the entire third column with the heading 'Total.'
15. Table 2 and its elaboration in the Results Section: Clarify the rationale for correlating the 'information desire' component with other components. If there's a specific reason, this could be explained in the introduction, results, or discussion sections. Otherwise, other components could be correlated with each other too. The second sentence in the discussion section also mentions about 'information requirement'. I was wondering, if this 'information requirement' could be rephrased, rather than just repeating the same jargon from the anxiety tool. It would be meaningful, if it is rephrased.
16. Results, Preoperative Anxiety Section, last sentence: Mention the strength of the correlation, whether it's strong, moderate, or weak.
17. Discussion, 1st Paragraph, 2nd sentence: Eliminate the restatement of 'median/mean score' in this context.
18. Discussion, 1st Paragraph, last sentence: Specify the reference (comparison) group when discussing the '2.5 times greater risk than...'.
19. Time frame when patients were assessed: In the Discussion section, 2nd paragraph, 2nd last sentence, you have mentioned about the time frame when patients were assessed. This is important. If this time duration is also mentioned in the method section of the abstract, it would be more meaningful to readers. 

Is the work clearly and accurately presented and does it cite the current literature?

Partly

If applicable, is the statistical analysis and its interpretation appropriate?

I cannot comment. A qualified statistician is required.

Are all the source data underlying the results available to ensure full reproducibility?

Partly

Is the study design appropriate and is the work technically sound?

Partly

Are the conclusions drawn adequately supported by the results?

Yes

Are sufficient details of methods and analysis provided to allow replication by others?

Yes

Reviewer Expertise:

I am an academic faculty and clinical psychologist, with research interest in psychometrics and psychological intervention.

I confirm that I have read this submission and believe that I have an appropriate level of expertise to confirm that it is of an acceptable scientific standard, however I have significant reservations, as outlined above.

# Response

**Published:** 2023-12-08

**Authors:** Bishnu Deep Pathak

**Affiliations:** Internal Medicine, Nepalese Army Institute of Health Sciences, Kathmandu, Bagmati, Nepal

Dear Reviewer,

 Thank you for reviewing our article. We have made necessary changes in the manuscript as appropriate and as suggested by you. We hope you will kindly consider our article for further approval. Below are the replies to your comments:

 1. Thank you for this comment. We have edited this in the manuscript.

 2. Thank you for this comment. We have added total number of cases in the abstract method section.

 3. Thank you for this comment. We have used only percentage in the result of abstract and have omitted the raw numbers.

 4. Thank you for this comment. We have edited it as you have suggested (eg. compared to reference groups like joing family, having past surgical history, etc).

 5. Thank you for this comment. The word 'rarely' has been replaced and the sentence has been rewritten appropriately.

 6. Thank you for this comment. We totally agree with your opinion that this is a cross sectional study. But, since we have used an inferential statistics in this study, this now becomes an analytical cross sectional study rather than just descriptive. 

 7. Thank you for this comment. We have used the term 'convenience sampling' as suggested by you. The word consecutive sampling has been replaced accordingly

 8. Thank you for this comment. The study referenced in sample size calculation looks very similar to our setting and in terms of design and methodology. Due to this reason, we found this study to be more appropriate for our sample size calculation. Please kindly consider it.

 9. Thank you for this comment. This sentence is intended to describe the different variables that we used in our questionnaire apart from APAIS tool while collecting data. And, these questions were prepared after extensive literature review by the investigators themselves. Almost all the studies included in the reference section were reviewed meticulously for the questionnaire. So, we think that referencing is not relevant in this part. Please kindly consider it.

 10. Thank you for this comment. We totally agree that lack of tranlation of APAIS tool into the native language is a major limitation of our study. But, as stated in the manuscript, the investigators had themselves asked the questions of this tool in a 'standard way.' Before doing this, the investigators had a discussion regarding how to ask each of the question to the patients in their native language. We had discussed about how much information to provide to the patients and also the way of asking each question. A special care was taken regarding the possibility of discrepancies that may arise while interviewing the cases. To avoid this error, all of the investigators involved in data collection had done a rehearsal beforehand as well. So, we had tried our best to minimize differences and possible bias in this scenario. But, still, this is a weak part of this study that needs to be rectified in future studies on this topic.

 11.Thank you for this comment. In our study, the margin of error has been taken as 7% and level of significance being 0.07.

 12, 13, 14. Thank you for these valuable comments. We have made edits in the manuscript as suggested by you

 15. Thank you for this comment. We have added a few sentences in the manuscript to address this. As described in the past literature, the information desired component is related to anxiety scores in the pre-operative period. It has been found that the higher the information score, the higher would be the anxiety level. In other words, those patients who were not properly informed about their surgical procedure, they would remain anxious and fearful regarding their surgery and would be expecting someone to provide them detail information leading to higher information component score in APAIS tool. Therefore, we used this statistical analysis in our study.

 16-19. Thank you for these valuable comments. We have made necessary changes in the manuscript as suggested.

# Reviewer report: Reviewer response for version 1

**DOI:** 10.5256/f1000research.149457.r210143

**Published:** 2023-10-17

**Authors:** Khagendra Kafle

**Affiliations:** 1 Department of Psychiatry, Chitwan Medical College Teaching Hospital (CMCTH), Chitwan, Nepal

The research topic is very relevant in the Nepalese context because there are very few studies done in this field. The methodology is sound. The tools used are fine. I have some comments on this article which I have mentioned below.  

1. Abstract: The background section can be rewritten. It should be shorter. It should normally contain what is known about the research topic and what is to be known about it (or the research objective) in 2-4 sentences.
2. There is a discrepancy about the included samples: elective surgeries (mentioned under study setting) or major elective surgeries (mentioned under sampling).
3. APAIS being a self-rated instrument, it should be in the native language of the research participants. Less education of the participants may not be the proper justification for not translating/validating it into the Nepali language. This is a major limitation of this study.
4. There is a discrepancy in the sampling technique mentioned in the article. It is mentioned as consecutive sampling at one place and convenience sampling in another place. Please correct it.

Is the work clearly and accurately presented and does it cite the current literature?

Yes

If applicable, is the statistical analysis and its interpretation appropriate?

Yes

Are all the source data underlying the results available to ensure full reproducibility?

Yes

Is the study design appropriate and is the work technically sound?

Yes

Are the conclusions drawn adequately supported by the results?

Yes

Are sufficient details of methods and analysis provided to allow replication by others?

Yes

Reviewer Expertise:

Psychiatry

I confirm that I have read this submission and believe that I have an appropriate level of expertise to confirm that it is of an acceptable scientific standard.

# Response

**Published:** 2023-12-08

**Authors:** Bishnu Deep Pathak

**Affiliations:** Internal Medicine, Nepalese Army Institute of Health Sciences, Kathmandu, Bagmati, Nepal

Dear Reviewer,

 Thank you for reviewing our article. We have made the necessary changes in the manuscript as suggested by you and as appropriate. We agree that the lack of translation of the APAIS tool in the native language is a major limitation of our study and hence we think that future studies on this topic need to rectify this aspect. Regarding sampling, we have used the term 'convenience sampling' throughout the text. We have tried to make our abstract as concise as possible.

 Thank you.
